# Development and psychometric properties of the Cognitive Distortions Questionnaire for Adolescents (CD-Quest-T)

**DOI:** 10.47626/2237-6089-2021-0214

**Published:** 2023-03-07

**Authors:** Claudia Luísa Sena Gomes de Souza, Pedro Paulo Pires, Isabela S. L. Couto, Nina S. S. M. de Vasconcelos, Igor G. Menezes, Irismar Reis de Oliveira

**Affiliations:** 1 Universidade Federal da Bahia Salvador BA Brazil Universidade Federal da Bahia, Salvador, BA, Brazil.; 2 Universidade Federal do Rio de Janeiro Rio de Janeiro RJ Brazil Universidade Federal do Rio de Janeiro, Rio de Janeiro, RJ, Brazil.; 3 University of Hull Hull United Kingdom University of Hull, Hull, United Kingdom.

**Keywords:** Psychometrics, cognitive distortions, adolescents, questionnaires

## Abstract

**Introduction:**

The Cognitive Distortions Questionnaire (CD-Quest) is an instrument that identifies logical errors or cognitive distortions and is used in trial-based cognitive therapy (TBCT). However, it had previously only been available for adults.

**Objectives:**

To develop and validate a version of the CD-Quest for teens (CD-Quest-T) aged 11 to 17 years and test its psychometric properties.

**Method:**

A total of 299 schoolchildren participated in the investigation. After content validity was assessed, the language was adapted for the target age group, and the length of the instrument was reduced to eight items (from the initial 15). Five cognitive therapists analyzed the content and structure of the items. Finally, to investigate the construct validity of the CD-Quest-T, the instrument was divided into a full scale and two subscales, which measure the frequency of the distortions and the intensity attributed to them, respectively.

**Results:**

The overall internal consistency of the scale was α = 0.77, whereas subscale indices were α = 0.75 for the frequency scale and α = 0.73 for the intensity scale. Results from exploratory factor analysis and concurrent validity analysis indicated that the CD-Quest-T items have good psychometric properties and generate scores reliably.

**Conclusion:**

The psychometric properties of the CD-Quest-T demonstrate its adequacy for measurement of cognitive distortions in adolescents.

## Introduction

One of the intervention methods used in cognitive-behavioral therapy (CBT) consists of addressing automatic thoughts (ATs), which are non-deliberate, spontaneous, quick, and pre-conscious cognitions. ATs are closely related to health because they directly affect emotions and behaviors. Psychological disorders follow specific cognitive patterns, maintained by dysfunctional core beliefs (CBs). When activated, these can generate dysfunctional emotions and behaviors, potentially impairing the person’s mental health and, consequently, their quality of life.^1,2^

According to Beck,^3^ events can be perceived distortedly. These perceptions are called cognitive distortions and, in excess, they can lead to emotional difficulties or exacerbate mental disorders, such as depression and anxiety.

The ways that adolescents interpret their life experiences shape their emotional reactions and behaviors. When a person’s dysfunctional CBs influence these ideas, logical errors or cognitive distortions can occur intensely and frequently. There is therefore a need for instruments to identify and quantify possible cognitive distortions in this age group. The objective is to find ways to prevent activation of dysfunctional CBs, since they may cause more problematic information processing and, consequently, impair adolescents’ healthy functioning (Table 1).^4,5^

**Table 1:** Original Cognitive Distortions Questionnaire (CD-Quest) items, indicating those modified for or excluded from the Cognitive Distortions Questionnaire for Adolescents (CD-Quest-T)

Original CD-Quest items	Items modified for CD-Quest-T	Items not included in CD-Quest-T
1. Dichotomous thinking	1. All-or-nothing thinking	4. Emotional reasoning
2. Fortune telling	2. Fortune telling	6. Magnification/Minimization
3. Discounting positives	3. Discounting positives	7. Selective abstraction
4. Emotional reasoning	4. Labeling	10. Personalization
5. Labeling	5. Mind reading	11. “Should” statements
6. Magnification/minimization	6. Overgeneralizing	14. What if...?
7. Selective abstraction	7. Jumping to conclusions	15. Unfair comparisons
8. Mind reading	8. Blaming	
9. Overgeneralization		
10. Personalization		
11. “Should” statements		
12. Jumping to conclusions		
13. Blaming		
14. What if...?		
15. Unfair comparisons		

There are specific instruments for measuring ATs in the current scientific literature. One of these is the Cognitive Distortions Questionnaire (CD-Quest), which is aimed at the general population and is mainly used in trial-based cognitive therapy (TBCT).^6^ It is also designed to identify cognitive errors to help patients in their daily task of identifying cognitive distortions and relating them to emotions and behaviors. It provides therapists with a quantitative measure for use in clinical follow-up.^6^ The psychometric properties of the CD-Quest have been evaluated in Brazil,^7^ the United States,^8,9^ Australia,^10^ Turkey,^11^ and China.^12^

Two studies have been conducted in Brazil to assess the psychometric properties of the adult version of the CD-Quest (one published^7^ and another still under consideration for publication). The first of these^7^ enrolled 184 university students and reported excellent supporting evidence for all stages of validation of the instrument. Factor analysis confirmed a unidimensional model and internal consistency measured with Cronbach’s α was 0.85. Moreover, concurrent validity analysis showed that the CD-Quest had moderate correlations with the Beck Depression Inventory (BDI) and the Beck Anxiety Inventory (BAI) (r = 0.65 and r = 0.52 respectively), and test-retest reliability was high (intraclass correlation coefficient [ICC] = 0.87). The second Brazilian study enrolled 197 university students and showed good internal consistency, with Cronbach’s α = 0.90 (non-published data; Seixas C, Vieira R, Pires PP, Butlerc RM, De Oliveira IR, Cognitive Distortions Questionnaire (CD-Quest): validation and psychometric properties in a sample of Brazilian undergraduate students, submitted 2020). This study also found a unidimensional factor solution, which corroborated the initial findings and showed that the CD-Quest is suitable for measuring cognitive distortions in the Brazilian population.

In the United States, Morrison et al.^8^ analyzed the psychometric properties of the CD-Quest administered to 906 students at a public university. They found excellent internal consistency with Cronbach’s α = 0.88. That study also identified a single factor and good psychometric properties, showing strong evidence of convergent validity with AT measures (e.g., the Automatic Thoughts Questionnaire-Revised [ATQ-R]),^13^ psychopathology measures (e.g., the Anxiety Sensitivity Index-3 [ASI-3]),^14^ and general functioning measures (e.g., the Quality of Life Inventory [QOLI]).^15^ Discriminant validity was confirmed against the Levenson Self-Report Psychopathology Scale (LSRP).^16^ Also, in the United States, Kaplan et al.^9^ conducted a study to assess the psychometric properties of CD-Quest in 216 patients diagnosed with social anxiety disorder (SAD). Once more, they observed excellent internal consistency, with Cronbach’s α = 0.91, and demonstrated a unidimensional structure using exploratory factor analysis (EFA) and confirmatory factor analysis.

In Australia, Kostoglou et al.^10^ validated the CD-Quest with a sample of 127 university students. Their study observed adequate internal consistency, with Cronbach’s α = 0.80, and significant positive correlations between the CD-Quest and both the ATQ^13^ and the Depression Anxiety Stress Scales (DASS).^17^ They also confirmed the questionnaire’s convergent and discriminant validity in the population studied.

Batmaz et al.^11^ studied the CD-Quest in clinical settings in two cities in Turkey. Their research was conducted with a sample of 269 patients and yielded excellent results for internal consistency with Cronbach’s α = 0.93, good test-retest reliability (*r *= 0.90), a unidimensional factor structure, and evidence of convergent and discriminant validity.

The reliability and validity of the Chinese version of the CD-Quest were recently confirmed in a sample of 239 Chinese college students. These results also suggested a unidimensional factor structure. Excellent internal consistency was demonstrated, with Cronbach’s α = 0.94, and test-retest reliability was 0.93.^12^

A literature review conducted for the present study did not find any questionnaires with characteristics equivalent to those of the CD-Quest-T. The one that came closest was the Children’s Negative Cognitive Error Questionnaire (CNCEQ), developed by Leitenberg et al.^18^ and revised by Messer et al.^19^

The CD-Quest-T is an adaptation of the original adult version of the CD-Quest,^6^ which identifies both the frequency and intensity with which cognitive distortions occur during the week. It is an initial self-monitoring instrument that helps in the patient’s psychoeducation and in understanding the distortions and errors a person may make in response to situations they experience. Therefore, the objective of this study was to develop the adolescent version of the CD-Quest, adapting it to this age group’s language.

## Method

This study was conducted in two phases, as described below. The objective of the first phase was to adapt the CD-Quest for adolescents and the objective of the second phase was to analyze the psychometric properties of the CD-Quest-T (Table 2).

**Table 2:** Measures of sampling adequacy (MSA) and Kaiser-Meyer-Olkin (KMO) values from the factor analysis of the items from the Cognitive Distortions Questionnaire for Adolescents (CD-Quest-T)

Item	MSA
1. All-or-nothing	0.87
2. Fortune telling	0.86
3. Discounting positives	0.80
4. Labeling	0.83
5. Mind reading	0.82
6. Overgeneralizing	0.83
7. Jumping to conclusions	0.87
8. Blaming	0.82
KMO	0.83

### Phase 1 – Adaptation of the CD-Quest-T

The data for development of the CD-Quest-T were collected using Survey Monkey (www.surveymonkey.com). Questions on cognitive distortions were previously sent via e-mail to cognitive therapists in Brazil using a list maintained by the Brazilian Federation of Cognitive Therapies (https://www.fbtc.org.br) and to therapists around the world through the Academy of Cognitive and Behavioral Therapies listserv (ACBT) (www.academyofct.org).

The therapists who participated (FBTC = 69; ACBT = 16) chose the five most common items among the adolescents they see from the list of 15 distortions on the adult version of the CD-Quest. They also suggested likely examples of cognitive distortions they dealt with in these patients’ therapeutic processes. Based on the therapists’ answers, the eight cognitive distortions most often found in adolescents were selected, along with their respective examples. The idea was to reduce the number of items from the original instrument from 15 to eight, making this a shorter version with more suitable language for identifying such distortions in this age group (Table 3).

**Table 3:** - Factor analysis of the individual items from the Cognitive Distortions Questionnaire for Adolescents (CD-Quest-T)

Item	Factor 1*	h2	Correlation item X result
1. All-or-nothing	0.38	0.14	0.86
2. Fortune telling	0.53	0.28	0.72
3. Discounting positives	0.35	0.12	0.88
4. Labeling	0.64	0.41	0.59
5. Mind reading	0.69	0.48	0.52
6. Overgeneralizing	0.65	0.42	0.58
7. Jumping to conclusions	0.62	0.41	0.59
8. Blaming	0.47	0.22	0.78

h2 = communality (common variance).

* Factor loadings.

After the CD-Quest-T had been designed, it was sent to five people who were not involved in the previous survey, and who agreed to accept the role of experts to assess the quality of item development. They were asked four questions about the items on the instrument: 1) Does each item adequately assess cognitive distortions? 2) Can they clearly evaluate the frequency and intensity of distortions? 3) Is the language adequate for adolescents? 4) Would you suggest any changes (e.g., inclusion or exclusion of items)?

In this phase, the experts evaluated the questionnaire based on definitions and items specified on an answer sheet developed for this purpose. The expected level of agreement on the content was between 70 and 100%.^20^

### Phase 2 – Assessment of the psychometric properties of the CD-Quest-T

#### Sample

The sample comprised 299 schoolchildren; 277 (92.6%) from two public schools and 22 (7.3%) from a private school, all in Salvador, Bahia, Brazil. Ages ranged from 11 to 17 years – encompassing the age group proposed in the study to adapt the CD-Quest-T. This equates to the sixth to ninth grades and 156 (52.1%) of the children were female and 143 (47.8%) were male. The sample was chosen according to the children’s schedules.

#### Instruments

Besides the CD-Quest-T, two other scales were used in this study to provide information on criteria validity: the Children’s Depression Inventory* (*CDI)^21^ and the Screen for Child Anxiety Related Emotional Disorders – Revised (SCARED-R).^22^

The CDI was developed to assess depressive symptoms in childhood, adapted from the BDI^23^ and devised by Kovacs.^24^ It is used with children aged 7 to 15 years old. It is a self-administered instrument and the original version has 27 items. Twenty items were used in this study, distributed in blocks with three affirmative sentences, to identify with what intensity and frequency each situation took place. The Brazilian version of the CDI was translated, adapted, and standardized by Gouveia et al.^25^ in a study with a sample of 305 schoolchildren, aged 8 to 15 years, from public and private schools in João Pessoa, Paraíba, Brazil. This study reported an internal consistency coefficient of 0.81. Another study conducted in public and private schools in São Luís, Maranhão, Brazil, with a sample of 280 schoolchildren aged 9 to 17 years old assessing the psychometric properties of the CDI reported a higher internal consistency index, with a Cronbach’s α of 0.91.^21^

The SCARED-R is a questionnaire developed by Birmaher et al.^26^ to assess anxiety-related emotional problems or disorders in children and adolescents. The version used in this study has 41 items and assesses different dimensions of anxiety in the age group from 8 to 18 years old. The scale is divided into ten categories corresponding to the subdivisions of anxiety disorders in the DSM-IV. It uses a three-point response scale (0 = never or seldom, 1 = sometimes, 2 = frequently) indicating the frequency with which a child has experienced the symptoms observed in the previous three months.^22,26,27^ Isolan et al.^28^ conducted a study of the psychometric properties of the Brazilian version of the SCARED-R with a sample of 2,410 schoolchildren aged 9 to 18 years old, reporting a Cronbach’s α of 0.90 for the total scale, and a test-retest reliability for the total score of r = 0.68 and an ICC of 0.81.

It was not necessary to change the original structure to adapt the adult version of the CD-Quest-T for adolescents, only the language used. Also, the number of cognitive distortion items was reduced from 15 to 8. The intention was to give it an adequate size and format, making it more suitable for the target age group. Additionally, a questionnaire was administered to obtain sociodemographic data such as age, school grade, and gender.

#### Procedure

Data on the adaptation of the CD-Quest-T were collected at three schools from July 2015 to July 2016, in 50-minute classes during school hours. This study is part of a larger study investigating preventive use of trial-based cognitive training (TBCTr) in schools.^29^ The intervention for that study comprised 18 group sessions developed for this age group and involving TBCTr, so the questions on the CD-Quest-T were explained to the schoolchildren at the end of one of the meetings related to cognitive distortions.

Three mental health professionals were available in the classroom to provide explanations if the schoolchildren had any doubts when filling out the CD-Quest-T. The schoolchildren had to identify their cognitive distortions, circling the number that corresponded to the frequency with which each had occurred in the previous week and the intensity with which they had believed in them at the time they occurred.

#### Data analysis

The CD-Quest-T’s psychometric properties were analyzed using R software. In a first phase, the content validity index (CVI) was estimated for the total score items. The minimum CVI defined as necessary for the items was as recommended by Almanasreh et al.^30^ – i.e., preferably 100%, while a value below 0.80 would indicate that the item needed to be either changed or removed. An EFA was conducted to assess construct validity. First, the factorability of the data was verified by estimating the Kaiser-Meyer-Olkin (KMO) index, for which the results are considered minimally adequate, beginning at 0.60. Individual measures of sampling adequacy (MSA) indexes for the items were also estimated. Bartlett’s sphericity test was used in this phase to confirm the adequacy of the correlation matrix, for which the items must present minimum collinearity. For the factor loadings presented, r > 0.30 was considered the minimum necessary value for retention of each item.^20^

Correlations between scales were used to test the CD-Quest-T’s concurrent validity against the SCARED-R and the CDI. Cronbach’s alpha (α) coefficients were estimated for the internal consistency and reliability analysis of the scales, adopting a reference value of α > 0.70, for coefficients calculated separately for the total CD-Quest-T score and for its frequency and intensity subscales.^31^

## Ethical considerations

The project was submitted to the research ethics committee at the Maternidade Climério de Oliveira, and approved under number 966.202. All the procedures adopted in this study comply with the ethics guidelines stipulated in Brazilian National Health Council Resolution 466/2012.^32^ All of the schoolchildren signed an assent form prior to participation, and their parents or legal guardians signed a consent form.

## Results

### Content validity

The first part of this study consisted of adapting the CD-Quest-T based on the results of the questions sent to the cognitive therapists. These therapists indicated which five of the 15 cognitive distortions on the adult CD-Quest they considered most common and frequent among adolescents. Their answers were used to identify the eight cognitive distortions most cited, which were selected for the adaptation.

The main characteristics of this version of the CD-Quest-T are the lower number of items (covering eight cognitive distortions), the appropriate language (which was made more straightforward for the age group), and changes to the examples for each cognitive distortion. The intention was to make the questionnaire easier to answer and as self-explanatory as possible so the adolescents could relate to the examples cited. These characteristics of the new instrument, which has a similar format to the adult version, were achieved by making changes to the definitions and examples provided.

The eight cognitive distortions selected were: 1. all-or-nothing thinking; 2. fortune-telling; 3. discounting positives; 4. labeling; 5. mind reading; 6. overgeneralizing; 7. jumping to conclusions; and 8. blaming. An example of the changes made to the text of the CD-Quest can be seen in the first distortion, dichotomous thinking (also known as all-or-nothing, black-and-white, or polarized), which was originally defined as follows: “I see events only in terms of ‘either this or that,’ placing them in two extreme categories, instead of a continuum” (examples: “I made a mistake, so my performance was a failure.” “I ate more than I had intended to, so I ruined my whole diet.”). In the CD-Quest-T version, using simpler language, the first distortion, all-or-nothing thinking, is as follows: “I see events and people as ‘all or nothing’ or ‘black or white,’ and don’t consider the gray areas.” The examples describe more adolescent-relevant situations, such as: “If I don’t get an A, this means I failed my test”; “Either I’m able to study all the subject matter, or I might as well not even try”; and “My Mom didn’t give me the present I wanted. She doesn’t like me.”

One of the experts who analyzed the adapted version was a psychometrics specialist and four were cognitive-behavioral therapists. All of them agreed that each item presented was related to its corresponding dimension and they did not propose any changes regarding the intended objective of the instrument. They also considered the language was adequate for the age group addressed in the study. The experts’ consensus on their evaluation and descriptive analysis of the questionnaire was that each item would be represented by a CVI exceeding 0.80. The design and content of the items in the CD-Quest-T achieved a CVI of 1.00 between the experts, indicating optimal quality and it was not therefore necessary to remove any of the items or modify the original structure.

### EFA

**Figure 1:**
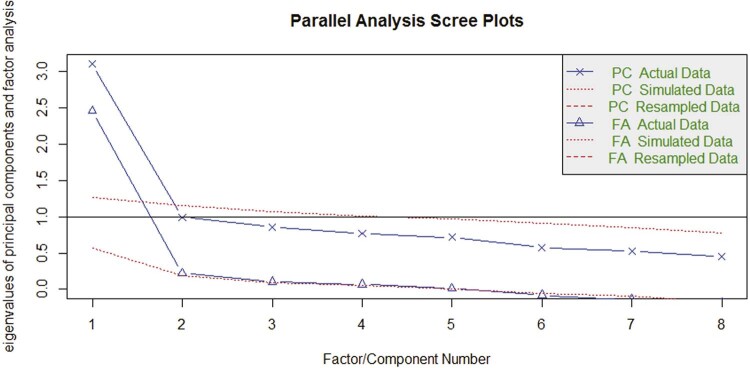
Curve inclined to almost 90 degrees, confirming that a single factor is enough.

Factor analysis was used to test the scale’s dimensionality. The preliminary results indicated that the data has sampling adequacy, with a KMO of 0.83, which suggests it was feasible to analyze the data using EFA. The result of Bartlett’s sphericity test (x^2^ = 27.113; p < 0.001) was significant, showing that the correlation matrix tends to be mostly different from the identity matrix (Figure 1).

The EFA results support a unidimensional solution with factor loadings ranging from 0.35 to 0.69. The commonalities (h2) ranged from 0.12 to 0.48, and the correlation between each item and the total varied from 0.52 to 0.86 for all of the questions on the CD-Quest-T, indicating good correlations. The proportion of variance explained was 31% for a single factor model. The adequacy of this number of factors was verified with a scree plot, confirming that a single factor was adequate.^31^

### Concurrent validity

The concurrent validity of the CD-Quest-T was confirmed against the SCARED-R and the CDI. Adopting r > 0.30 as a cutoff, significant correlation coefficients were found. The correlation with the total SCARED-R score was r = 0.31 (p < 0.01) and the correlation with the total CDI score was r = 0.30. The correlations between the frequency subscale score and the SCARED-R and CDI scores were r = 0.41 and r = 0.46 respectively. In turn, the intensity subscale had correlations of r = 0.43 for SCARED-R and r = 0.40 for CDI.

### Internal consistency analysis

Cronbach’s α for internal consistency was calculated separately for the total score and for the frequency and intensity subscales. A Cronbach’s α of 0.77 was found for the internal consistency of the entire questionnaire, while Cronbach’s alphas of 0.75 and 0.73 were found for the frequency and intensity scales respectively, indicating good and adequate internal consistency in all measurement levels.^30^ It should be noted that excluding any one item would not have resulted in variation in the overall Cronbach’s α value (Table 4).

**Table 4:** Cronbach’s alpha

Item	Total Cronbach’s alpha if one item is excluded
1. All-or-nothing	0.76
2. Fortune telling	0.74
3. Discounting positives	0.77
4. Labeling	0.73
5. Mind reading	0.72
6. Overgeneralizing	0.73
7. Jumping to conclusions	0.73
8. Blaming	0.76

## Discussion

The present study aimed to adapt the CD-Quest for adolescents (Brazilian Portuguese and English versions available as online-only supplementary material), and analyze its psychometric properties, gathering evidence to support its content and construct validity.

The factor analysis indicated that the instrument represents a single dimension, according to analysis of its psychometric properties, with satisfactory factor loadings. However, some of the item commonalities were relatively problematic, which may be due to the heterogeneity of cognitive distortions. The CD-Quest-T also presented evidence of concurrent validity, indicating that its scores were correlated with both the SCARED and the CDI. It is to be expected that cognitive distortions would have significant positive correlations, indicating presence of symptoms of anxiety and depression. There is evidence to suggest that adolescents with anxious or depressive disorders have distorted cognition, sustained by dysfunctional CBs about themselves, the world, and other people.^3,5,^^33^

Unfortunately, as the sample underwent a therapeutic intervention, a test-retest study was not conducted as part of the reliability phase of the psychometric analysis of this sample, which could be considered a limitation of this study. Consequently, the results do not provide information on the temporal stability of the CD-Quest-T scores. Nonetheless, its internal consistency results were favorable.

Given the hypotheses formulated, conclusion of development of the CD-Quest-T items and evaluation of its psychometric properties yielded satisfactory evidence of the functioning of its items and scores. The results were similar to those found in the literature regarding the adult version of the CD-Quest as a unidimensional instrument.^7,9,11^ However, future studies are needed to evaluate the scores’ stability and also the instrument’s sensitivity to clinical conditions. More information on the nomological network would enrich understanding of how informative the CD-Quest-T can be when interacting with other variables related to mental health.

An assessment of content validity was conducted in this study as an essential step in ascertaining that the CD-Quest-T’s format and content are adequate for the target population. Regarding information on its psychometric properties, we also provided multiple analyses showing evidence of both construct and criterion validity, understanding that validity is a perspective on multiple cumulative levels.

This study has a few limitations. First, it was conducted with schoolchildren from only one Brazilian city. Second, it did not involve a clinical sample, which suggests future investigations need to be conducted to analyze the psychometric properties of the CD-Quest-T in different settings and populations. Third, the study is limited by a lack of information on other types of reliability assessment, such as internal consistency, denoting that more information is needed on the stability of the scores over time, especially if the scale is intended to monitor change during therapy. Future studies should enlarge the known nomological network to provide more information on the functioning of the CD-Quest-T scores and their relationships with other clinical variables. Finally, future efforts should aim to compose test norms for score interpretation while also testing the potential use of the CD-Quest-T for detecting different psychological conditions, which could be provided by an ROC curve analysis in a clinical sample.

In summary, the results of the present study provide preliminary psychometric underpinnings for use of the CD-Quest-T as a brief self-report measure of cognitive distortions, showing that it is a valid and reliable instrument for assessing such cognitions in adolescents.
