# Supplementary material for: Development and psychometric properties of the Cognitive Distortions Questionnaire for Adolescents (CD-Quest-T)
Source: Trends Psychiatry Psychother. 2023 Mar 7;45:e20210214. doi: 10.47626/2237-6089-2021-0214 (PMC10164402; doi:10.47626/2237-6089-2021-0214)
Supplement: Supplementary file 1 [file 2238-0019-trends-45-e20210214-suppl.pdf]

Supplementary Material S1

Portuguese Version (CD-Quest-T)

Questionário de Distorções Cognitivas CD-Quest

Versão para Adolescentes

Irismar Reis de Oliveira

Nome: .... **Data:**.............................. **Serie:**...............................

Leia com atenção e faça um círculo em torno do número que melhor descreve quantas vezes e em que intensidade os erros ou distorções cognitivas ocorreram durante esta semana. Ao avaliar cada distorção cognitiva, por favor, indique quanto você acreditou nela no momento em que ela aconteceu (e não quanto você acredita agora) e com que frequência ela aconteceu durante esta semana.

DURANTE ESTA SEMANA, PERCEBI QUE ESTAVA PENSANDO DA SEGUINTE FORMA:

1. **Pensamento tudo-ou-nada:** Vejo os acontecimentos e as pessoas como "tudo ou nada" ou "oito ou oitenta". Não considero o que está no meio.
EXEMPLOS: "Se não tiro 10, isso significa que fracassei na prova". "Ou consigo estudar todo o assunto, ou é melhor nem tentar." "Minha mãe não me deu o presente que eu queria. Ela não gosta de mim."

Frequência: / Intensidade:	Não aconteceu	Aconteceu 1 a 2 dias na semana	Aconteceu 3 a 5 dias na semana	Aconteceu o tempo todo, 6 a 7 dias na semana
Acreditei...	0			
Um pouco		1	2	3
Médio		2	3	4
Muito		3	4	5

2. **Previsão do futuro:** Adivinho o futuro, como se tivesse uma bola de cristal, geralmente prevendo o pior.
EXEMPLOS: "Vou ficar nervoso e me dar mal na prova". "A diretora me chamou para ir à secretaria. Ela vai brigar comigo". "A festa vai ser muito ruim".

Frequência: / Intensidade:	Não aconteceu	Aconteceu 1 a 2 dias na semana	Aconteceu 3 a 5 dias na semana	Aconteceu o tempo todo, 6 a 7 dias na semana
Acreditei...	0			
Um pouco		1	2	3
Médio		2	3	4
Muito		3	4	5

3. **Desqualificação do positivo:** Desmereço ou diminuo o valor das coisas boas e positivas que acontecem comigo.
EXEMPLOS: "Só tirei nota boa na prova porque estava fácil." "Minha mãe ficou contente porque eu a ajudei, mas isso não foi mais do que minha obrigação." "Passar de ano não foi grande coisa. Qualquer um consegue."

Frequência: / Intensidade:	Não aconteceu	Aconteceu 1 a 2 dias na semana	Aconteceu 3 a 5 dias na semana	Aconteceu o tempo todo, 6 a 7 dias na semana
Acreditei...	0			
Um pouco		1	2	3
Médio		2	3	4
Muito		3	4	5

4. **Rotulação:** Coloco um carimbo, um rótulo ou uma etiqueta geralmente negativo em mim ou nos outros.
EXEMPLOS: "Maria é metida." "Paulo é antipático." "Sou burro." "Sou um fracasso."

**Frequência:** / **Intensidade:**	**Não aconteceu**	**Aconteceu 1 a 2 dias na semana**	**Aconteceu 3 a 5 dias na semana**	**Aconteceu o tempo todo, 6 a 7 dias na semana**
**Acreditei...**	0			
**Um pouco**		1	2	3
**Médio**		2	3	4
**Muito**		3	4	5

5. **Leitura mental:** Acredito que posso adivinhar o que as pessoas estão pensando ou que elas podem adivinhar meus pensamentos.
EXEMPLOS: "Alice não me ligou porque não quer sair comigo." "Pela cara do professor, tirei nota baixa." "Jane passou e nem me olhou. Deve estar zangada comigo."

**Frequência:** / **Intensidade:**	**Não aconteceu**	**Aconteceu 1 a 2 dias na semana**	**Aconteceu 3 a 5 dias na semana**	**Aconteceu o tempo todo, 6 a 7 dias na semana**
**Acreditei...**	0			
**Um pouco**		1	2	3
**Médio**		2	3	4
**Muito**		3	4	5

6. **Generalização:** Generalizo e exagero as coisas usando palavras como "sempre", "nunca", "tudo", nada", "só", etc.
EXEMPLOS: "Ninguém gosta de mim." "Nada comigo dá certo". "Meus pais nunca me entendem".

**Frequência:** / **Intensidade:**	**Não aconteceu**	**Aconteceu 1 a 2 dias na semana**	**Aconteceu 3 a 5 dias na semana**	**Aconteceu o tempo todo, 6 a 7 dias na semana**
**Acreditei...**	0			
**Um pouco**		1	2	3
**Médio**		2	3	4
**Muito**		3	4	5

7. **Conclusões precipitadas:** Tiro conclusão precipitada e faço as coisas de forma apressada, sem observar melhor o que está se passando.
EXEMPLOS: "Fui mal na primeira unidade. Não adianta continuar, vou abandonar o colégio." "Se não respondeu a minha mensagem é porque não se importa comigo." "Se olhou para minha namorada, é porque está paquerando ela."

**Frequência:** / **Intensidade:**	**Não aconteceu**	**Aconteceu 1 a 2 dias na semana**	**Aconteceu 3 a 5 dias na semana**	**Aconteceu o tempo todo, 6 a 7 dias na semana**
**Acreditei...**	0			
**Um pouco**		1	2	3
**Médio**		2	3	4
**Muito**		3	4	5

8. **Culpar:** Culpo as pessoas pelo que me acontece, não considerando minha responsabilidade no acontecimento.
EXEMPLOS: "Meus pais são os únicos culpados por minha infelicidade." "Tirei nota baixa por culpa da professora." "Cheguei atrasado porque minha mãe não me acordou."

**Frequência:** / **Intensidade:**	**Não aconteceu**	**Aconteceu 1 a 2 dias na semana**	**Aconteceu 3 a 5 dias na semana**	**Aconteceu o tempo todo, 6 a 7 dias na semana**
**Acreditei...**	0			
**Um pouco**		1	2	3
**Médio**		2	3	4
**Muito**		3	4	5

Supplementary Material S2

English Version (CD-Quest-T)

Cognitive Distortions Questionnaire

Version for Teenagers (CD-Quest-T)

Copyright (c) 2015

Irismar Reis de Oliveira

Many thoughts go through our minds. Many of these thoughts are correct, however, many others are inaccurate.

For this reason they are called thinking errors or cognitive distortions.

Take this example: Paul is a good student who handed in his essay, which the teacher corrected. She made few corrections.

Paul became nervous and thought: "The teacher didn't like it. If the essay were good, she wouldn't have made any corrections."

To Paul, there are only two conditions: either the essay is good, or it is bad. This type of thinking error is known as "all or nothing."

Since Paul had this thought on Friday and Saturday (two days), and Paul believed it a lot, he circled number 3 in the second column of the chart below.

1. **All-or-nothing thinking:** I see events and people as "all or nothing" or "black or white," and don't consider the gray areas.

EXAMPLES: "If I don't get an A, this means I failed my test." "Either I'm able to study all the subject matter, or I might as well not even try."

"My Mom didn't give me the present I wanted. She doesn't like me."

Paul's example*: My essay is really bad. If it were a good one, my teacher wouldn't have made any corrections.*

Frequency:<br>Intensity:	Didn't Happen	It happened 1 to 2 days this week	It happened 3 to 5 days this week	It happened all the time, 6 to 7 days this week
**I believed it...**	0			
**A little**		1	2	3
**Somewhat**		2	3	4
**A lot**		3	4	5

Please turn the page and evaluate how you think.

Cognitive Distortions Questionnaire

Version for Teenagers (CD-Quest-T)

Irismar Reis de Oliveira

Name: .... Date:.............................. Grade:..............................

Read carefully and circle the number that best describes how many times the cognitive distortions happened and how intense they were <u>during this week.</u> When evaluating each cognitive distortion, please indicate <u>how much</u> you believed it at the time it happened (and not how much you believe it now) and <u>how often</u> it happened during this week.

DURING THIS WEEK, I NOTICED THAT I WAS THINKING IN THE FOLLOWING MANNER:

1. **All-or-nothing thinking:** I see events and people as "all or nothing" or "black or white," and don't consider the gray areas.

EXAMPLES: "If I don't get an A, this means I failed my test." "Either I'm able to study all the subject matter, or I might as well not even try."

"My Mom didn't give me the present I wanted. She doesn't like me."

Frequency: / Intensity:	Didn't Happen	It happened 1 to 2 days this week	It happened 3 to 5 days this week	It happened all the time, 6 to 7 days this week
**I believed it...**	0			
**A little**		1	2	3
**Somewhat**		2	3	4
**A lot**		3	4	5

2. **Fortune-telling:** I guess the future, as if I had a crystal ball, usually predicting the worst.

EXAMPLES: "I'll be nervous and do poorly on the test." "The principle called me to the office. I'm in trouble." "The party will be awful."

Frequency: / Intensity:	Didn't Happen	It happened 1 to 2 days this week	It happened 3 to 5 days this week	It happened all the time, 6 to 7 days this week
**I believed it...**	0			
**A little**		1	2	3
**Somewhat**		2	3	4
**A lot**		3	4	5

http://doi.org/10.47626/2237-6089-2021-0214

3**. Discounting positives:** I discredit or decrease the merit of the good and positive things that happen to me.

EXAMPLES: "I only got a good grade on the test because it was easy." "My Mom was happy because I helped her, but it was nothing more than my duty."

"Passing my final exams wasn't a big deal; anyone can do that."

**Frequency:**<br><br>**Intensity:**	**Didn't Happen**	**It happened 1 to 2 days this week**	**It happened 3 to 5 days this week**	**It happened all the time,**<br><br>**6 to 7 days this week**
**I believed it...**	0			
**A little**		1	2	3
**Somewhat**		2	3	4
**A lot**		3	4	5

4. **Labeling:** I put a stamp, a label or a tag, usually negative, on myself or others.

EXAMPLES: "Maria is a snob." "Paulo is rude." "I'm stupid." "I'm a failure."

**Frequency:**<br><br>**Intensity:**	**Didn't Happen**	**It happened 1 to 2 days this week**	**It happened 3 to 5 days this week**	**It happened all the time,**<br><br>**6 to 7 days this week**
**I believed it...**	0			
**A little**		1	2	3
**Somewhat**		2	3	4
**A lot**		3	4	5

5. **Mind reading:** I believe I can guess what people are thinking or that they can guess my thoughts.

EXAMPLES: "Alice didn't call me because she doesn't want to go out with me." "From the looks on my teacher's face, I got a bad grade."

"Jane walked by and didn't even look my way. She must be mad at me."

**Frequency:**<br><br>**Intensity:**	**Didn't Happen**	**It happened 1 to 2 days this week**	**It happened 3 to 5 days this week**	**It happened all the time,**<br><br>**6 to 7 days this week**
**I believed it...**	0			
**A little**		1	2	3
**Somewhat**		2	3	4
**A lot**		3	4	5

6. **Overgeneralizing:** I generalize and exaggerate things by using words like "always," "never," "all," "nothing," "only," etc.

EXAMPLES: "Nobody likes me." "I can't do anything right." "My parents never understand me."

**Frequency:** **Intensity:**	**Didn't Happen**	**It happened 1 to 2 days this week**	**It happened 3 to 5 days this week**	**It happened all the time, 6 to 7 days this week**
**I believed it...**	0			
**A little**		1	2	3
**Somewhat**		2	3	4
**A lot**		3	4	5

7. **Jumping to conclusions:** I jump to conclusions and do things hastily, without taking a better look at what's going on.

EXAMPLES: "I did badly in the first quarter. No use continuing, I'm going to quit school." "If he didn't reply to my message, it's because he doesn't care about me." "If he looked at my girlfriend, it's because he is flirting with her."

**Frequency:** **Intensity:**	**Didn't Happen**	**It happened 1 to 2 days this week**	**It happened 3 to 5 days this week**	**It happened all the time, 6 to 7 days this week**
**I believed it...**	0			
**A little**		1	2	3
**Somewhat**		2	3	4
**A lot**		3	4	5

8. **Blaming:** I blame others for what happens to me, without considering my responsibility in the situation.

EXAMPLES: "My parents are the only ones to blame for my unhappiness." "It's the teacher's fault that I got a poor grade." "I arrived late because my mom didn't wake me up."

**Frequency:** **Intensity:**	**Didn't Happen**	**It happened 1 to 2 days this week**	**It happened 3 to 5 days this week**	**It happened all the time, 6 to 7 days this week**
**I believed it...**	0			
**A little**		1	2	3
**Somewhat**		2	3	4
**A lot**		3	4	5
